# An Improved Method for High Quality Metagenomics DNA Extraction from Human and Environmental Samples

**DOI:** 10.1038/srep26775

**Published:** 2016-05-31

**Authors:** Satyabrata Bag, Bipasa Saha, Ojasvi Mehta, D. Anbumani, Naveen Kumar, Mayanka Dayal, Archana Pant, Pawan Kumar, Shruti Saxena, Kristine H. Allin, Torben Hansen, Manimozhiyan Arumugam, Henrik Vestergaard, Oluf Pedersen, Verima Pereira, Philip Abraham, Reva Tripathi, Nitya Wadhwa, Shinjini Bhatnagar, Visvanathan Gnana Prakash, Venkatesan Radha, R. M. Anjana, V. Mohan, Kiyoshi Takeda, Takashi Kurakawa, G. Balakrish Nair, Bhabatosh Das

**Affiliations:** 1 Molecular Genetics Laboratory, Centre for Human Microbial Ecology, Translational Health Science and Technology Institute, NCR Biotech Science Cluster, Faridabad, India; 2 Section of Metabolic Genetics, Novo Nordisk Foundation Center for Basic Metabolic Research, University of Copenhagen, Copenhagen, Denmark; 3 Department of Gastroenterology, P.D. Hinduja Hospital and Medical Research Centre, Mumbai, India; 4 Department of Obstetrics and Gynaecology, Maulana Azad Medical College, New Delhi, India; 5 Pediatric Biology Center, Translational Health Science and Technology institute, NCR Biotech Science Cluster, Faridabad, India; 6 Madras Diabetes Research Foundation, No. 4, Conran Smith Road, Gopalapuram, Chennai, India; 7 Dept. of Microbiology and Immunology, Graduate School of Medicine, Osaka University, Japan

## Abstract

To explore the natural microbial community of any ecosystems by high-resolution molecular approaches including next generation sequencing, it is extremely important to develop a sensitive and reproducible DNA extraction method that facilitate isolation of microbial DNA of sufficient purity and quantity from culturable and uncultured microbial species living in that environment. Proper lysis of heterogeneous community microbial cells without damaging their genomes is a major challenge. In this study, we have developed an improved method for extraction of community DNA from different environmental and human origin samples. We introduced a combination of physical, chemical and mechanical lysis methods for proper lysis of microbial inhabitants. The community microbial DNA was precipitated by using salt and organic solvent. Both the quality and quantity of isolated DNA was compared with the existing methodologies and the supremacy of our method was confirmed. Maximum recovery of genomic DNA in the absence of substantial amount of impurities made the method convenient for nucleic acid extraction. The nucleic acids obtained using this method are suitable for different downstream applications. This improved method has been named as the THSTI method to depict the Institute where the method was developed.

Efficient extraction of high-quality, high molecular weight (HMW) community genomic DNA from limited amount of human origin or environmental samples carrying diverse microbial species is the key challenge for cutting edge downstream applications like next generation DNA sequencing (NGS). The NGS technology is often used to explore the identity and abundance of culturable and uncultured microbial species in its natural community and to decode the microbial genomes to investigate its functional repertoires. For such different applications including shotgun metagenomics it is very important to extract HMW community genomic DNA. Different microbes present in diverse ecosystems have different types of cell wall and cell membranes, which enclose their cytoplasm and genomic contents (Fig. 1). Harsh sample treatment could affect DNA quality, while mild process may cause partial lysis particularly for the classes of bacteria carrying thick layers of peptidoglycan. Therefore, it is important to optimize the cell lysis methods to obtain genomic DNA from abundant as well as rare representatives of each taxonomic groups possessing different thickness of cell wall and different layer of cell membranes with different embedded components casing their genomic contents.

Lyses of microbial cells expose their genomic DNA to different cellular and extracellular molecules including different type of nucleases. Despite its inert nature, double stranded DNA is physically fragile and highly susceptible to exo- and endonucleases, active forms of which are widely present in the matrix of most of the environmental and human samples analyzed in this study. Therefore, it is important to inactivate all the nucleases in lysis solution by incorporating strong denaturing agents or chemicals that chelate residual metallic ions from the suspension. Although, several commercial kits are now available to extract DNA from human and environmental samples, most of which uses silica-based column where DNA adsorb selectively to a stationary solid phase at high pH and high salt concentration. The major disadvantage for most of the commercial kits is insufficient recovery of genomic DNA from marginal amount of clinical or environmental samples. Furthermore, different DNA extraction kits have different biases, which can produce dramatically different results for the same sample^1^. Several laboratories working on metagenomics reported different methods of community DNA extraction depending on the type of samples they used for analysis^2^,^3^,^4^,^5^,^6^,^7^,^8^. Recently, International Human Microbiome Standards (IHMS) launched a guideline for standard operating procedures to optimize community DNA extraction methods from human fecal samples (http://www.microbiome-standards.org). So far, no attempt has been taken to develop a gold standard for community DNA extraction from both human and environmental origin samples.

In this study, we developed a highly sensitive method, by combining physical, mechanical and chemical lysis approaches, to isolate community bacterial DNA from different human and environmental samples (Fig. 2). All the selected samples harbor culturable and uncultured bacteria belonging to closely or distantly related taxonomic groups and having different thickness of cell wall and different layer of cell membranes (Fig. 1). We compared both the quality and quantity of isolated community DNA with existing methodologies and observed that this approach worked best compared to currently available approaches. The isolated DNAs are suitable for all types of high-resolution downstream applications including shotgun metagenomics sequencing where HMW genomic DNA is preferable.

## Results and Discussion

### Spheroplast formation and DNA isolation

**Figure 1:**
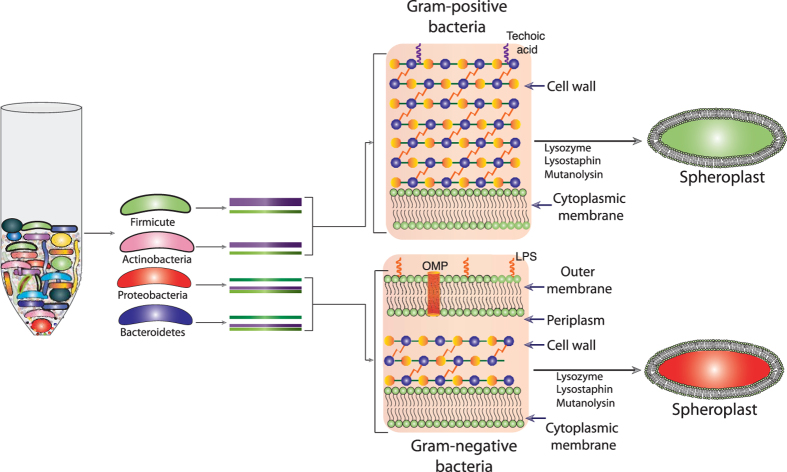
Diverse microbial species living in different ecosystems have different cell membranes and different types of cell wall encasing their cytoplasm. Outermembrane cover and cell wall can be removed by treating the microbial community with specific enzymes that use polymer or transpeptide bridge of cell wall as their substrate.

Both, environmental and human samples contain large numbers of microbial cells belonging to different phyla and they are reasonably heterogeneous in terms of their genomic contents, morphology and architecture of their cell wall (Fig. 1). To obtain sufficient amount of quality community DNA from Gram-positive and Gram-negative bacterial cells, it is important to preprocess the samples before adding lysis reagents. In this study, we used three different enzymes lysozyme, lysostaphin and mutanolysin that target either 1,4-beta glycoside-linkages or transpeptide bond in Gram-positive and Gram-negative bacterial cell wall and help in spheroplast formation. Spheroplast is highly susceptible to lysis reagents and labile to mechanical and physical forces.

**Figure 2:**
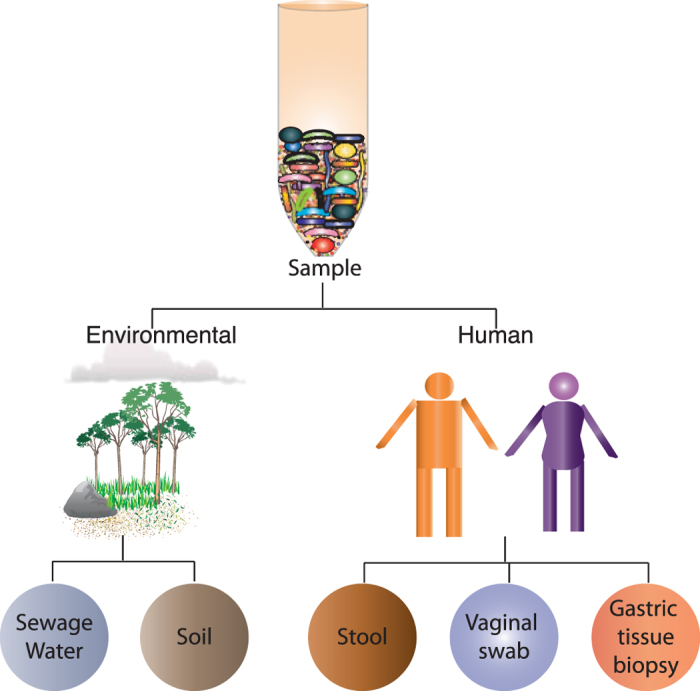
Different environmental and human samples used in this study to isolate community DNA from culturable and uncultured microbial residents.

**Figure 3:**
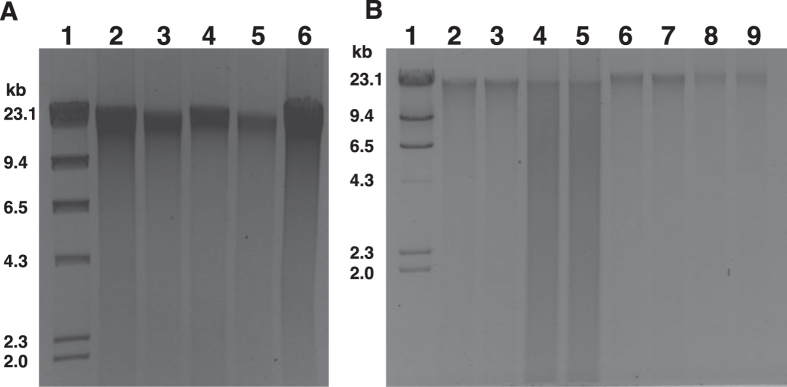
Agarose gel electrophoresis of microbial genomic DNA isolated from environmental and human samples. Genomic DNA was electrophoresed on a 0.8% agarose gel, stained with ethidium bromide and photographed in a gel imaging system. (**A**) Genomic DNA isolated by THSTI method. Lane 1: Lambda genomic DNA digested with restriction endonuclease *Hind*III; lane 2: Genomic DNA isolated from Sewage water *(SW),* lane 3: Genomic DNA isolated from soil sample, lane 4: Genomic DNA isolated from stool, lane 5: Genomic DNA isolated from vaginal swab (VS), lane 6: Genomic DNA isolated from gastric tissue biopsy (GTB) sample. (**B**) Genomic DNA isolated from equal amount of samples using commercial kits or automated liquid handling system. Lane 1: Lambda genomic DNA digested with restriction endonuclease *Hind*III; Lane 2–3: Genomic DNA isolated from stool samples using commercial kit. Lane 4–5: Genomic DNA isolated from GTB samples using commercial kit. Lane 6–7: Genomic DNA isolated from stool DNA samples using automated liquid handling system. Lane 8–9: Genomic DNA isolated from VS samples using automated liquid handling system.

For lysis, first we treated the spheroplast with Guanidinium thiocyanate (GITC) to disrupt the bacterial cell membrane and inactivate nucleases and other enzymes. Combining mechanical (bead beating) and thermal (heat) forces enabled final lysis. The recovery and quality of the isolated DNAs were confirmed by running the samples on agarose gel (Fig. 3). We used both environmental and human samples (Fig. 2), containing diverse range of bacterial species including Gram-positive and Gram-negative bacteria possessing different types of cell wall, to confirm the suitability of the same method in wide range of samples. We successfully isolated reasonably good amount of quality DNA from all the tested samples (Fig. 3 and Table 1). DNA yield was typically ~1–109 μg, depending on the initial sample size and the way the sample was stored (Table 1). Total yield of DNA irrespective of the sample types was always higher in THSTI method compare to Kit and ALHS methods (Table 1). Average size of the DNA fragments recovered by THSTI method was ~20 kb (Fig. 3).

### Assessment of the quality of isolated DNA

Both the quality and quantity of isolated DNA were assessed by measuring the absorbance at 260 and 280 nm wavelengths (Table 1) and by visualizing extracted community DNA on agarose gel (Fig. 3). Most of the isolated DNA samples had OD_260_/OD_280_ ratio in between ~1.6 and ~1.9 except the genomic DNA isolated from soil sample (Table 1). We further confirmed the quality of isolated DNA by visualizing all the samples on 0.8% agarose gel containing DNA-intercalating agent ethidium bromide. Although, the gel electrophoresis is not very sensitive to measure the quantity of DNA but this is useful to analyze the stable RNA contamination, short fragment DNA contamination, and also shown the average size of isolated DNA. It is important to note that in THSTI and kit methods nucleic acids were treated with RNase to remove stable RNA while in automated liquid handling system the RNAse treatment step was absent. Thus, in terms of quality of DNA, the present method is free of from other nucleic acid impurities.

### Comparison of current method with available DNA isolation kits and automated nucleic acid extraction system

Several methods have been described for community microbial DNA extraction from human and environmental origin samples^7^,^8^,^9^,^10^,^11^,^12^,^13^. We compared the quality and quantity of DNA obtained from equal amount of same samples for all, except gastric tissue biopsy, using DNA isolation kit (Qiagen, Germany), and automated nucleic acid extraction system (MagNA pure, Roche Diagnostics, Swizerland). We observed that, when the tested samples, like stool specimen, contained large numbers of bacterial species, both automated nucleic acid extraction system and kit method could recover adequate amount of quality DNA for downstream applications. However, both the methods are not efficient to recover sufficient amount of DNA from low amount of microbial cells including vaginal swabs, where bacterial number was limited (Fig. 3 and Table 1). In contrast, the method developed in this study efficiently recoverd sufficient amount of genomic DNA even in samples with limited amount of bacterial cells (Fig. 3 and Table 2).

### Suitability of isolated DNA in different downstream applications

**Figure 4:**
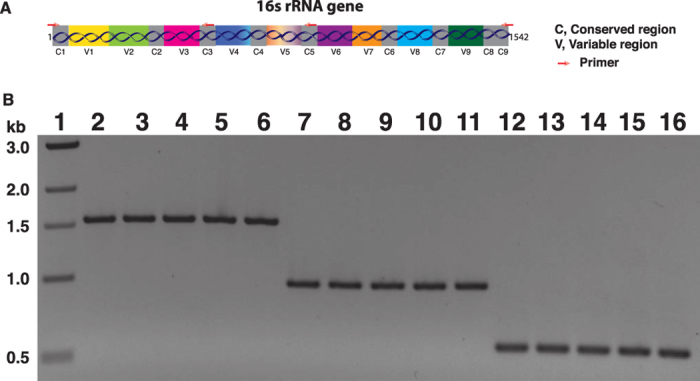
PCR amplification of 16S rRNA gene from community DNA isolated from environmental and human origin samples. (**A**) Organization of conserved and variable regions of 16S rRNA gene. Small arrows indicate different primers used in this study to amplify partial or complete 16S rRNA gene. C denotes conserved while V indicates variable. (**B**) PCR amplification of complete or partial 16S rRNA gene using primers tagged with or without different barcode and adaptor sequences for 454 GS FLX+ pyrosequencer. Genomic DNA isolated both from environmental (SW, Soil) or human samples (Stool, VS, GTB) were used as template. Lane 1: 1-kb DNA ladder; Lane 2–6: complete 16S rRNA gene amplicons from SW, Soil, Stool, VS, GTB, respectively; Lane 7–11: V1-V5 region amplicons of 16S rRNA gene of SW, Soil, Stool, VS, GTB, respectively; Lane 12–16: V1-V3 region amplicons of 16S rRNA gene of SW, Soil, Stool, VS, GTB, respectively.

**Figure 5:**
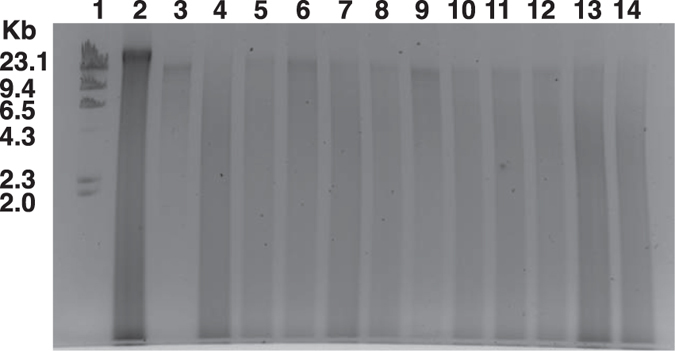
Restriction endonuclease (*Eco*RI) digestion of genomic DNA isolated from environmental and human origin samples using kit, ALHS and THSTI methods. Lane 1, Lambda genomic DNA digested with restriction endonuclease *Hind*III; Lane 2, undigested genomic DNA isolated from stool sample; Lane 3–5, *Eco*RI digested stool genomic DNA sample isolated by kit, ALHS and THSTI methods, respectively; Lane 6–8: *Eco*RI digested HVS sample isolated by kit, ALHS and THSTI methods, respectively; Lane 9–11: *Eco*RI digested genomic DNA of soil sample isolated by kit, ALHS and THSTI methods, respectively; Lane 12–14: *Eco*RI digested genomic DNA of sewage water sample isolated by kit, ALHS and THSTI methods, respectively.

**Figure 6:**
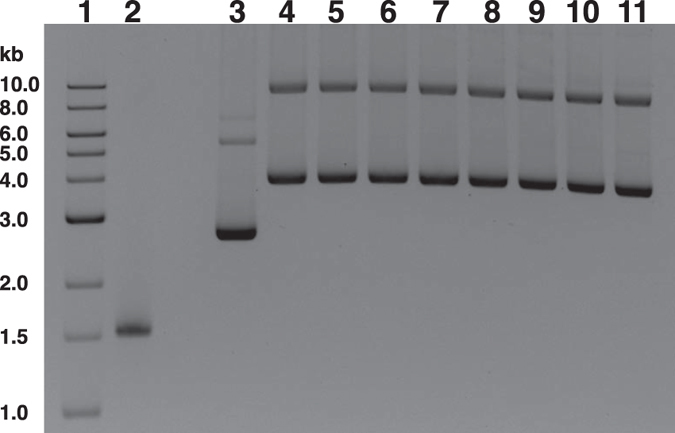
PCR amplification and cloning of complete 16S rRNA gene of sewage water samples. Lane 1: 1-kb DNA ladder. Lane 2: 16S rRNA gene PCR amplicon. Lane 3: Cloning vector pCR2.1. Lane 4–11: Cloning vector containing complete 16S rRNA gene isolated from eight randomly selected clones.

To assure the quality of isolated nucleic acid, the samples were used for different downstream applications including PCR amplification (Fig. 4), restriction digestion (Fig. 5), cloning and sequencing of PCR products (Fig. 6). The PCR amplification of complete and partial 16S rRNA gene of bacterial DNA was done by using set of primer tagging with or without NGS specific adaptor and barcode sequences. The adaptor was selected based on the recommendation of 454 GS FLX+ pyrosequencing platform (Table 3). We used different NGS primers specific for C1, C3 and C5 and C9 regions of 16S rRNA gene (Fig. 4 and Table 3). Sufficient amount of desired amplicon from each set of amplification reaction confirmed the suitability of isolated DNA for NGS application (Fig. 4). The complete 16S rRNA genes were amplified from the sewage water, soil, stool, GTB and vaginal swabs genomic DNA and subset of them were used for cloning and sequencing reactions. Among thousands of clones obtained during cloning of 16S rRNA gene, few of them were randomly picked up for plasmid isolation. Eight representative recombinant clones of 16S rRNA gene amplified from sewage water DNA are shown (Fig. 6). Insert of subset of plasmids were sequenced in a capillary sequencer using universal M13F and/or M13R primers. Identity of 16S rRNA genes amplified from DNA sample of sewage water, soil, stool, GTB and vaginal swabs were examined by using NCBI BLASTN program (https://blast.ncbi.nlm.nih.gov/Blast.cgi?PAGE_TYPE=BlastSearch) database. Although the sample size was small (n = 36), still we have identified multiple Gram-positive and Gram-negative bacterial species in different samples belonging to different bacterial classes (Table 4). Restriction digestions of subset of DNA samples were done using type II restriction endonuclease *Eco*RI (Fig. 5). Complete digestion of genomic DNA indicates absence of inhibitory compounds, possibly, in the isolated DNA samples.

## Conclusion

The method reported in this study is very efficient and economic to isolate community bacterial DNA from minimal amount of human and environmental samples. The quality and quantity of extracted DNA are suitable for various downstream applications including restriction enzyme digestion, PCR amplification using sequencing adaptor and barcode tagged primers used for NGS reactions. Compared to testified two methods, kit and automated nucleic acid extraction system, the recovery of community DNA in THSTI method is substantially higher. A limitation of the present method is the duration for extraction of DNA from the sample. This can be afforded, considering the quality, quantity and suitability of the isolated DNA for subsequent downstream applications.

## Methods and Materials

### Samples

Sewage water and soil, two environmental samples used for this study, were collected from the National Capital Region, India. Stool samples were obtained from healthy adult volunteers. Gastric biopsy samples were obtained from Hinduja Hospital and Medical Research Centre, Mumbai, India. Vaginal swab samples were obtained from Department of Obstetrics and Gynecology, *Maulana Azad Medical College, New Delhi, India and* Pediatric Biology Center, Translational Health Science and Technology Institute, NCR Biotech Science Cluster, Faridabad, India. The human origin samples were collected after receiving approval from THSTI ethics committee and informed consent from the study subjects. Recombinant DNA works were carried out in “accordance” with the approved guidelines of THSTI biosafety committee. All other experimental protocols used in this study were carried out in “accordance” with the relevant guidelines and standard operating procedure (SOP) of Centre for human microbial ecology (CHME).

### Enzymes

Lysozyme (10 mg/ml), mutanolysin (25 KU/ml) and lysostaphin (4 KU/ml) were used for removal of cell wall from Gram-positive and Gram-negative bacterial cells. All three enzymes were purchased from Sigma-Aldrich, USA. Both mRNA and stable RNA species were removed from the pool of nucleic acids by treating the samples with RNase (10 mg/ml).

### Buffers

Tris-HCl (IM, pH 8.0 and pH 7.5) and Phosphate buffer (0.1 M, pH 8.0) were used to re-suspend the nucleic acids.

### Other reagents

Following reagents were used at different stages of sample processing and DNA isolation: EDTA (0.5 M, pH 8.0), NaCl (5 M), PVPP (Mol wt 40,000), Guanidine thiocyanate (4 M), Sodium-acetate (3 M, pH 5.2), Potassium acetate (5 M, pH 5.2), N-Laurylsarcosine (10%), Glass beads (2.5 mm), Zirconia beads (0.1 mm), Ethanol (96%), Hydrochloric acid (HCl), Sterile deionized water (H_2_O). All the chemicals used in this study were purchased from Sigma-Aldrich, USA.

### Glass Beads processing

The glass beads are very useful to detach microbes from the matrix of collected samples. 2.5 mm glass beads are suitable for bacterial cells. First, the glass beads (Biospec USA) were kept in 1.0% Triton-X solution for 30 minutes at room temperature and then washed 6–7 times with water. The washed beads were kept in an incubator over night at 55 °C. Beads were autoclaved before use.

### Preparation of 0.1 mm Zirconia beads

First, the 0.1 mm Zirconia beads (Biospec USA) were washed with 1% Triton-X solution. All the detergent was removed by vigorous shaking and washing the beads 7–8 times in milliQ water until it does not foam anymore. The beads were resuspended in milliQ water and sterilized by autoclaving at 121 °C for 15 min.

### Pre-processing and cell lysis

First, fresh or freeze stored environmental (1 gm soil, 35 ml SW) or human samples (200 mg stool, 1 HVS, 1–5 mg GTB) carrying sufficient numbers of bacterial cells were transferred into a pre-chilled 2 ml microcentrifuge tube (MCT) and resuspended in 200 μl 50 mM Tris-1 mM EDTA buffer (pH 8.0). Since, all the samples contain both microbial cells and extracellular matrix like, mucin or undigested food particles, it is important to detach the microbes for adequate access of buffering agents and enzymes, used for spheroplast formation. With this aim, we added 4 glass beads (2.5 mm) and vortexed continuously for 1 min or until the sample was thoroughly homogenized. Then the glass beads were removed from the suspension by transferring supernatant into a fresh tube and enzyme cocktail containing 50 μl lysozyme (10 mg/ml); 6 μl mutanolysin (25 KU/ml), and 3 μl lysostaphin (4 KU/ml) was added. The cell suspension was incubated for 1 hour at 37 °C to remove cell wall from bacterial cells.

Lysis of microbial cells was done by combining chemical, physical and mechanical approaches. First, 250 μl Guanidine thiocyanate (4 M) was added and mixed gently for 45 seconds. Then, 300 μl 10% N-Lauryl sarcosine was added and incubated for 10 minutes at 37 °C in a vortex mixer (Thermomixer, Eppendorf, Germany) with mild shaking (300 rpm). After short incubation, the tubes were transferred into a pre-warmed water bath and incubated at 70 °C for 1 hour. Mechanical lysis was done in a bead beater using 0.1 mm zirconia beads (BioSpec, USA). Around 300 mg of zirconia beads was added to the suspension and cell lysis was done by mechanical disruption using SpeedMill PLUS bead beater (Analytical Jena, Germany). Beating was done in two cycles (30 seconds each). Total program time for bacteria was 2 minutes. After completion of bead beating, 15 mg Polyvinylpolypyrrolidone (PVPP) was added to the suspension and mixed well by gentle vortexing of the sample. To remove the added beads, PVPP and all other cell debris, the suspension was spun down at 14000 rcf for 5 minutes in a microcentrifuge (5427R, Eppendorf, Germany).

### Organic extraction and precipitation of nucleic acids

The supernatant was transferred into a fresh MCT. The pellet was washed with 500 μl Tris (50 mM)-EDTA(20 mM)-NaCl(100 mM)-PVPP(1%) and the supernatants were pooled. The genomic DNA was precipitated from the supernatant by adding two volumes of 96% ethanol. The organic solvent was mixed gently for one minute and kept five minutes at room temperature and the nucleic acids were recovered by centrifugation at maximum speed, 14000 rcf, for 10 minutes at 4 °C in a microcentrifuge. The supernatant was removed by mild aspiration and keeping the tube in an inverted position on adsorbent paper to let the fluid drain away. The pelleted nucleic acids were dried for 10–15 minutes at room temperature.

### Removal of RNA and purification of genomic DNA

To remove all the RNA species that are present in the nucleic acid preparation, the pellet was dissolved in 450 μl phosphate buffer supplemented with 50 μl 3 M-potassium acetate. The pellet was dissolved by pipetting and incubated on ice for 90 minutes. The tube was removed from ice and 2 μl RNase (10 mg/ml) was added and placed in a heating block (37 °C) for 30 minutes. The suspension was supplemented with 50 μl sodium-acetate (3 M) and 1 ml of ice-cold 96% ethanol. The DNA was precipitated by centrifugation at 14000 rcf for 10 minutes at 4 °C. To remove the excess salts, the pellet was washed with 70% ice-cold ethanol. The pellet was dried at room temperature and re-suspended in 200 μl Tris (10 mM)-EDTA (1 mM) buffer (pH 8.0) and dissolved DNA was stored at 4 °C.

### PCR amplification and cloning of community 16S rRNA gene

PCR amplification of 16S rRNA gene for targeted metagenomics study was done using adaptor and barcode labeled conserved region specific primers and DNA free Q5® High-fidelity DNA polymerase (NEB, USA). Amplification was done in 50 μl reaction volume with 1–10 ng of template DNA and following the reaction conditions: 98 °C-2 minute (1 cycle), 98 °C-20 seconds, 50 °C-30 seconds, 72 °C-45 seconds (30 cycle), 72 °C-7 minute (1 cycle). The PCR products were electrophoresed on a 1% agarose gel, stained with ethidium bromide and photographed using a gel imaging system (Alphaimager, USA). PCR amplified 16S rRNA gene products from sewage water, soil, stool, GTB and vaginal swab samples were purified and cloned into pCR2.1 cloning vector and subsets of samples were sequenced in a capillary sequencer using vector specific M13F and/or M13R primers.

## Highlights

- Sensitive method to isolate community bacterial DNA from different human origin and environmental samples.
- Efficient recovery and high purity of isolated DNA made this method attractive for high-resolution molecular applications.
- Would be gold standard for wide range of studies including environmental and clinical samples.
- Very economic compared to kits and automated DNA extraction methods.

## Box 1

*Lysozyme*, well known antimicrobial peptide, is a lytic enzyme that disrupts bacterial cell walls by catalyzing hydrolysis of 1,4-beta glycoside-linkages between N-acetylmuramic acid and N-acetyl-D-glucosamine residues present in the peptidoglycan layer.

*Lysostaphin*, a 27 KDa glycylglycine endopeptidase, used as antimicrobial agent against Gram-positive bacteria^14^. The endopeptidase works on the transpeptide bond of bacterial cell wall and removes the crosslinking peptide bridges.

*Mutanolysin* is a an N-acetylmuramidase that catalyzes the cleavage of β-N-acetylmuramyl-(1 → 4)-N-acetylglucosamine linkage of the Gram-positive bacterial cell wall^15^. Its N-terminal end carries enzymatic domain where the C-terminal moieties are involved in substrate recognition and binding to the unique cell wall polymers. The enzyme is preferably used in the formation of spheroplasts and isolation of DNA from bacterial culture.

### *Guanidinium thiocyanate*

(GITC) is a chaotropic agent, used as strong denaturant to isolate nucleic acids from viral particles and bacterial cells^16^. GITC is used to lyse cells and inactivate RNase and DNase, the enzymes that is present in all bacterial cells and degrade RNA and DNA, respectively.

*Sodium lauroyl sarcosinate*, an amphiphilic amino acid anionic surfactant comprising hydrophobic 12-carbon aliphatic chain and the hydrophilic carboxylate, most often used in nucleic acid isolation from bacterial cells^17^. It helps in lysis of host cells and removing protein and broken cell walls from the suspension.

### *Polyvinylpolypyrrolidone*

(PVPP) is an insoluble, cross-linked form of polyvinylpyrrolidone. PVPP helps to remove the host secondary metabolites and other phenolic impurities from aqueous solution.

### Isopropanol and Ethanol

Since, isopropanol is less volatile than ethanol and it co-precipitates simple sugars and salts with nucleic acids, precipitation of DNA with ice cold, 96% ethanol is preferable. DNA is a highly polar molecule, because of its negatively charged phosphate residues in the nucleotide backbone. The repulsive forces that arise because of the exposed phosphate group between the polynucleotide chains need to be neutralized for effective precipitation of DNA. In the presence of 70% ethanol and 300 mM Na^+^ ions, the negative charges of the polynucleotide chains are reduced to the point where the DNA precipitates. It is important to note that ethanol precipitation of DNA can only be done if the cations are available in sufficient amount.

## Additional Information

**How to cite this article**: Bag, S. *et al.* An Improved Method for High Quality Metagenomics DNA Extraction from Human and Environmental Samples. *Sci. Rep.* **6**, 26775; doi: 10.1038/srep26775 (2016).

## Figures and Tables

**Table 1:** Average concentration and total recovery of nucleic acids isolated from different environmental and human origin samples.

Sample	Method	Nucleic acid concn. (ng/μl)	Total recovery (ng)	260/280
Stool	THSTI	543.3 ± 187.26 (DNA)	108660 ± 37520 (DNA)	1.85 ± 0.06
Stool	Kit	202.29 ± 105.63 (DNA)	20229.23 ± 10563 (DNA)	1.94 ± 0.23
Stool	ALHS	113.38 ± 62.26 (DNA + RNA)	11338.46 ± 6226 (DNA + RNA)	1.67 ± 0.07
Vaginal Swab	THSTI	104.77 ± 39.61 (DNA)	20955.38 ± 7923.13 (DNA)	1.69 ± 0.12
Vaginal Swab	Kit	8.37 ± 5.66 (DNA)	836.15 ± 566.7 (DNA)	1.43 ± 0.58
Vaginal Swab	ALHS	22.79 ± 9.5 (DNA + RNA)	2279.23 ± 906.02 (DNA + RNA)	2.47 ± 1.01
Soil	THSTI	53.16 ± 36.77 (DNA)	10633.84 ± 10317.18 (DNA)	1.48 ± 0.041
Soil	Kit	66.02 ± 70.13 (DNA)	6602.30 ± 7014 (DNA)	1.16 ± 0.05
Soil	ALHS	93.91 ± 103.17 (DNA + RNA)	9391.53 ± 7355.84 (DNA + RNA)	1.44 ± 0.07
Sewage water	THSTI	79.24 ± 80.71 (DNA)	15849.23 ± 12190 (DNA)	1.71 ± 0.041
Sewage water	Kit	14.47 ± 5.72 (DNA)	1447.69 ± 572 (DNA)	1.68 ± 0.05
Sewage water	ALHS	98.74 ± 60.95 (DNA + RNA)	9874.61 ± 8071 (DNA + RNA)	2.14 ± 0.07
Gastric Tissue Biopsy	THSTI	53.9 (DNA)	10780 (DNA)	1.85
Gastric Tissue Biopsy	Kit	126.5 (DNA+RNA)	12650 (DNA+RNA)	1.4

In this study, 13 random samples from each category, except gastric tissue biopsy (n = 3), were used for comparative study. It is important to note that during nucleic acid extraction by the THSTI and Kit methods RNase treatment was included, while in the automated liquid handling system (ALHS) RNase treatment step is missing. Soil samples were heterogenous and maximum differences in DNA yield from similar amount of different samples were observed in each methods.

**Table 2:** Minimum number of bacterial cells needed to isolate detectable amount of nucleic acids by using genomic DNA isolation kit or THSTI methods.

CFU	10^9^	10^8^	10^7^	10^6^	10^5^	10^4^
Kit	6.27 μg	0.28 μg	0.03 μg	ND	ND	ND
THSTI	10.3 μg	1.25 μg	0.1 μg	0.03 μg	ND	ND

**Table 3:** Primers used in this study to amplify partial or complete 16S rRNA gene.

Name	Sequence (5′-3′)
130	GGCGGATCCAAGGAGGTGTTCCAGCCGC
139	GGCCTCGAGAGAGTTTGATCCTGGCTCAGG
27F	CCTATCCCCTGTGTGCCTTGGCAGTCTCAGAGAGTTTGATCCTGGCTCAG
534R	CCATCTCATCCCTGCGTGTCTCCGAC*TCAG*CACGCATTACCGCGGCTGCTGG
926R	CCATCTCATCCCTGCGTGTCTCCGAC*TCAG*CACGCCCGTCAATTCMTTTRAGT

Letter code: Bold font, Restriction enzyme binding sequence; Regular font, 16S rRNA gene specific sequence; Regular ubderline font, adaptor sequence for 454 GS FLX+ pyrosequencer; Italic font, Key sequence for454 GS FLX+ pyrosequencer; Bold underline font, barcode (MID) sequence.

**Table 4:** Dominant bacterial species identified in the sewage water (SW) samples, soil samples (SS), stool samples (GM), vaginal swabs (HVS) and gastric tissue biopsy samples (GTB).

Clone	Bacterial species	Systemic position	GenBank accession no.
GM01-Pc	*Prevotella copri*	Class-Bacteroidetes, Phylum- Bacteroidetes	KX057366
GM02-Pc	*Prevotella copri*	Class-Bacteroidetes, Phylum- Bacteroidetes	KX057367
GM03-Pc	*Prevotella copri*	Class-Bacteroidetes, Phylum- Bacteroidetes	KX057368
GM04-Pb	*Prevotellaceae bacterium*	Class-Bacteroidetes, Phylum- Bacteroidetes	KX057369
GM05-Pc	*Prevotella copri*	Class-Bacteroidetes, Phylum- Bacteroidetes	KX057370
GM06-Pc	*Prevotella copri*	Class-Bacteroidetes, Phylum- Bacteroidetes	KX057371
GM07-Pc	*Prevotella copri*	Class-Bacteroidetes, Phylum- Bacteroidetes	KX057372
HVS01-LCr	*Lactobacillus crispatus*	Class-Bacilli, Phylum-Firmicutes	KX057346
HVS02-LCr	*Lactobacillus crispatus*	Class-Bacilli, Phylum-Firmicutes	KX057347
HVS03-LCr	*Lactobacillus crispatus*	Class-Bacilli, Phylum-Firmicutes	KX057348
HVS04-LCr	*Lactobacillus crispatus*	Class-Bacilli, Phylum-Firmicutes	KX057349
HVS05-LCr	*Lactobacillus crispatus*	Class-Bacilli, Phylum-Firmicutes	KX057350
HVS06-LCr	*Lactobacillus crispatus*	Class-Bacilli, Phylum-Firmicutes	KX057350
HVS07-Ljn	*Lactobacillus jensenii*	Class-Bacilli, Phylum-Firmicutes	KX057352
HVS08-Lco	*Lactobacillus coleohominis*	Class-Bacilli, Phylum-Firmicutes	KX057353
HVS09-Lco	*Lactobacillus coleohominis*	Class-Bacilli, Phylum-Firmicutes	KX057354
HVS10-Lco	*Lactobacillus coleohominis*	Class-Bacilli, Phylum-Firmicutes	KX057355
HVS11-Lco	*Lactobacillus coleohominis*	Class-Bacilli, Phylum-Firmicutes	KX057356
HVS12-Lco	*Lactobacillus coleohominis*	Class-Bacilli, Phylum-Firmicutes	KX057357
GTB01-Gh	*Gemella haemolysans*	Class-Bacilli, Phylum-Firmicutes	KX057343
GTB02-Hp	*Helicobacter pylori*	Class-Epsilonproteobacteria, Phylum-Proteobacteria	KX057344
GTB03-Hp	*Helicobacter pylori*	Class-Epsilonproteobacteria, Phylum-Proteobacteria	KX057345
SW01-BP	Unculture betaproteobacterium	Class-Betaproteobacteria, Phylum-Proteobacteria	KX057358
SW02-RB	*Rhodobacterales bacterium*	Class-Alphaproteobacteria, Phylum-Proteobacteria	KX057359
SW03-Ac	*Actinobacterium sp.*	Class-Actinobacteria, Phylum- Actinobacteria	KX057360
SW04-Ar	*Arcobacter sp.*	Class-Epsilonproteobacteria, Phylum-Proteobacteria	KX057361
SW05-Mb	*Macromonas bipunctata*	Class-Betaproteobacteria, Phylum-Proteobacteria	KX057362
SW06-Ab	*Alcaligenaceae bacterium*	Class-Betaproteobacteria, Phylum-Proteobacteria	KX057363
SW07-Bs	*Bordetella sp.*	Class-Betaproteobacteria, Phylum-Proteobacteria	KX057364
SW08-Pa	*Pseudomonas aeruginosa*	Class-gammaproteobacteria, Phylum-Proteobacteria	KX057365
SS01-Bi	*Bacillus infantis*	Class-Bacilli Phylum-Firmicutes	KX129724
SS02-Rs	*Rhizobium sp.*	Class-Alphaproteobacteria Phylum-Proteobacteria	KX129725
SS03-Bs	*Bacillus sp.*	Class-Bacilli Phylum-Firmicutes	KX129726
SS04-Pt	*Psychroflexus sp.*	Class-Flavobacteriia Phylum-Bacteroidetes	KX129727
SS05-Fc	*Flavobacterium sp.*	Class-Flavobacteriia Phylum-Bacteroidetes	KX129728
SS06-Zp	*Gramella sp.*	Class-Flavobacteriia Phylum-Bacteroidetes	KX129729
